# Development and validation of a clinical nomogram for predicting cumulative live birth rate in ovarian endometrioma patients undergoing ethanol sclerotherapy and *in vitro* fertilization/intracytoplasmic sperm injection

**DOI:** 10.3389/fendo.2026.1738786

**Published:** 2026-02-06

**Authors:** Bowen Liu, Yamei Li, Qian Zhou, Feng Wang, Shuping Liu, Chahui Zhang, Jinjiang Huang, Weifen Deng, Yuhua Shi

**Affiliations:** 1 Guangdong Cardiovascular Institute, Guangdong Provincial People’s Hospital, Guangdong Academy of Medical Sciences, Guangzhou, China; 2 Reproductive Medicine Center, Shenzhen Hengsheng Hospital, Shenzhen, China; 3 Department of Obstetrics and Gynecology, Center for Reproductive Medicine, Nanfang Hospital, Southern Medical University, Guangzhou, China

**Keywords:** cumulative live birth rate, ethanol sclerotherapy, *in vitro* fertilization, nomogram, ovarian endometrioma, prediction model

## Abstract

**Background:**

Effective individualized tools to predict cumulative live birth rates are lacking for patients with ovarian endometriomas undergoing *In Vitro* Fertilization or Intracytoplasmic Sperm Injection after ultrasound-guided ethanol sclerotherapy. This study aimed to construct and validate a nomogram model for predicting the cumulative live birth rate in this specific population.

**Methods:**

This retrospective study analyzed the clinical data of 194 patients with ovarian endometriosis who underwent ethanol sclerotherapy followed by *In Vitro* Fertilization or Intracytoplasmic Sperm Injection between January 2020 and December 2024. All patients were randomly divided into training (n = 135) and validation (n = 59) cohorts in a 7:3 ratio. Independent risk factors for the cumulative live birth rate were identified through a comprehensive three-stage screening process that included univariate regression, Least Absolute Shrinkage and Selection Operator regression, and multivariate logistic regression analyses. The nomogram model was constructed using the selected variables, and its discrimination, calibration, and clinical utility were assessed using Receiver Operating Characteristic curves, calibration curves, and Decision Curve Analysis.

**Results:**

A total of 194 patients were included, with an overall cumulative live birth rate of 50.0% (97/194). Multivariate regression analysis identified four independent predictors of the cumulative live birth rate: previous live birth history, controlled ovarian hyperstimulation protocol, number of oocytes retrieved, and cyst diameter. The nomogram constructed using these variables exhibited good discriminatory ability in both the training and validation cohorts, with areas under the curve of 0.849 (95% Confidence Interval: 0.782-0.912) and 0.853 (95% Confidence Interval: 0.754-0.952), respectively. Bootstrap validation (500 iterations) confirmed the stability of the model. Calibration was acceptable (Hosmer-Lemeshow test, P>0.05), and decision curve analysis indicated a favorable net benefit across a threshold probability range of 10-50%, demonstrating its clinical utility.

**Conclusions:**

This study developed and validated a parsimonious nomogram model that accurately predicts the cumulative live birth rate in patients with ovarian endometriosis undergoing *In Vitro* Fertilization/Intracytoplasmic Sperm Injection after ethanol sclerotherapy. Based on four key predictors (previous live birth history, controlled ovarian hyperstimulation protocol, number of oocytes retrieved, and cyst diameter), the model demonstrates excellent and robust predictive performance. It serves as an intuitive and reliable clinical tool to support individualized counseling and shared treatment decision-making.

## 1 Introduction

Endometriosis (EMs) affects approximately 10% of reproductive-aged women and is a major cause of pelvic pain and infertility (1, 2). Ovarian endometriomas (OMAs) occur in 17–44% of cases and impair fertility through pelvic anatomical distortion, proinflammatory environments, and damage to adjacent ovarian tissue, leading to a reduced ovarian reserve (3, 4). Managing OMAs in fertility-seeking patients requires careful balancing of symptom control and ovarian preservation.

The stripping technique removes the healthy ovarian cortex alongside the cyst walls, substantially lowering postoperative anti-Müllerian hormone (AMH) levels and antral follicle count (AFC) (5, 6). This functional decline is particularly concerning *In Vitro* Fertilization/Intracytoplasmic Sperm Injection (IVF/ICSI) candidates, as a reduced ovarian reserve correlates with poor stimulation responses and decreased live birth rates (7).

Ultrasound-guided ethanol sclerotherapy (EST) has emerged as a minimally invasive alternative for the management of OMAs. This procedure involves cyst aspiration followed by ethanol instillation, which chemically ablates the cyst lining while preserving the ovarian reserve (8, 9). Meta-analyses reveal effective cyst reduction and pain relief, with AMH levels remaining stable or improving, in contrast to the significant decline observed post-cystectomy (10). The mechanism of action involves disruption of the cyst’s epithelial lining through the direct cytotoxic effects of ethanol, followed by inflammation and fibrosis, which ultimately leads to cyst obliteration (11). Unlike surgical excision, EST precisely targets OMAs without damaging the surrounding normal ovarian tissue, thereby better preserving the ovarian reserve and follicular pool (12). Systematic reviews have demonstrated that EST results in comparable or superior oocyte retrieval compared with laparoscopic cystectomy, with pregnancy rates ranging from 20% to 57% (8, 11). When performed before IVF/ICSI, EST may enhance reproductive outcomes by reducing local inflammation and supporting folliculogenesis (13).

Despite these advantages, predicting individual live birth outcomes remains challenging. IVF/ICSI success depends on the complex interactions among patient characteristics, disease factors, and treatment parameters (14). Although biomarkers such as AMH are routinely used, their predictive accuracy for live births remains limited (15). Nomograms have shown superior accuracy over single-variable models in reproductive medicine (16, 17); however, there has been no prediction of the cumulative live birth rate (CLBR) in patients with OMAs undergoing IVF/ICSI after EST.

This study sought to develop and validate a nomogram for predicting CLBR in OMAs who underwent EST prior to IVF/ICSI. We hypothesized that a multivariable model incorporating clinical, sonographic, and treatment variables would yield accurate individualized predictions to enhance patient counseling and inform clinical decision-making.

## 2 Materials and methods

### 2.1 Study design, ethical approval, and reporting guidelines

This retrospective cohort study was conducted at the Reproductive Medicine Center of Shenzhen Hengsheng Hospital in accordance with the established guidelines for observational research in the field of reproductive medicine. The study population consisted of women diagnosed with OMAs who underwent ultrasound-guided EST before their IVF/ICSI cycle between January 2020 and December 2024. The study protocol was approved by the hospital’s Institutional Review Board (HSYY2022-12-22) and adhered to the principles outlined in the Declaration of Helsinki for medical research involving human subjects. Owing to the retrospective design of the study, the requirement for informed consent was waived, and appropriate data anonymization procedures were implemented. The reporting of this manuscript adheres to the Transparent Reporting of a Multivariable Prediction Model for Individual Prognosis or Diagnosis (TRIPOD) statement for prediction model development and validation (18).

### 2.2 Participants

#### 2.2.1 Inclusion criteria

The eligibility for inclusion in the study was determined based on the following criteria: (1) female participants aged between 20 and 45 years at the time of ultrasound-guided EST; (2) a diagnosis of OMAs confirmed via transvaginal ultrasound, characterized by unilocular or multilocular cysts with homogeneous, low-level “ground-glass” echogenicity, with at least one cyst measuring 4 cm or larger in diameter; (3) receipt of EST as the primary treatment for OMAs, followed by the initiation of at least one complete IVF/ICSI cycle within 6 months; and (4) availability of comprehensive clinical data, including complete baseline characteristics, controlled ovarian hyperstimulation (COH) parameters, embryological outcomes, and follow-up records until the determination of pregnancy outcomes or cycle cancellation.

#### 2.2.2 Exclusion criteria

Patients were excluded from the study if they met any of the following criteria: (1) absence of more than 20% of key predictor variables; (2) history of ovarian surgery for OMAs or adnexal pathologies that compromise ovarian reserve; (3) severe male factor infertility; (4) known chromosomal abnormalities or genetic disorders in either partner; (5) concurrent malignancy or severe systemic disease; and (6) inability to follow up before the determination of pregnancy outcomes.

### 2.3 Outcome definitions

The primary outcome was the CLBR, defined as the delivery of a live-born infant with a gestational age of at least 28 weeks resulting from all fresh and frozen-thawed embryo transfers following the first completed oocyte retrieval cycle after EST (19). The CLBR was determined for each patient, considering embryo transfers until a live birth was achieved or all embryos from the initial cycle were utilized. The follow-up period extended from oocyte retrieval to either live birth or final embryo transfer, with a minimum duration of 12 months, to adequately capture reproductive outcomes.

### 2.4 Interventions

#### 2.4.1 Ultrasound-guided ethanol sclerotherapy

All EST procedures were conducted by reproductive endocrinologists with over five years of experience, adhering to a standardized institutional protocol informed by established international guidelines (20). A 17-gauge needle was used to puncture the endometrioma under transvaginal ultrasound guidance. Complete aspiration of the cyst contents was performed, and the aspirated volume and fluid characteristics were documented. Absolute ethanol (95%) was introduced into the cyst cavity, comprising 50–80% of the aspirated volume, with a maximum volume of 8 mL for 10–15 min. During this period, gentle agitation was employed to ensure optimal contact between the ethanol and the cyst wall. Subsequently, ethanol was aspirated, and the cavity was irrigated with saline to eliminate any residual sclerosants from the cavity. Patients were administered antibiotics and observed for 1–2 h before discharge. A follow-up transvaginal ultrasound was conducted at 4–6 weeks to evaluate cyst reduction and treatment efficacy before proceeding with IVF/ICSI cycles.

#### 2.4.2 *In vitro* fertilization/Intracytoplasmic sperm injection

COH protocols were selected according to current evidence-based guidelines and individualized based on patient characteristics (21, 22). Available protocols include ultra-long, long, antagonist, mild stimulation, progestin-primed ovarian stimulation (PPOS), and natural cycle approaches to ovarian stimulation. Protocol selection followed standard clinical practice, considering the patient’s age, ovarian reserve markers, and previous treatment responses (23). The starting dose of gonadotropins (recombinant Follicle-Stimulating Hormone (FSH) or human menopausal gonadotropin) was 150–300 IU daily. Dose adjustments were based on follicular development, which was monitored through transvaginal ultrasound and hormone tests. Final oocyte maturation was induced with human Chorionic Gonadotropin (hCG) or Gonadotropin-Releasing Hormone (GnRH) agonist when two dominant follicles reached a diameter of ≥ 18 mm. Transvaginal oocyte retrieval was performed 34–36 h after the trigger under ultrasound guidance. Fertilization was performed via conventional IVF or ICSI, based on semen parameters and history. Embryos were cultured to the cleavage (day 3) or blastocyst stage (day 5/6) and assessed for quality. Fresh embryo transfer was conducted, or viable embryos were cryopreserved for frozen embryo transfer (FET) cycles. Luteal support was provided using progesterone, according to the institutional protocols.

### 2.5 Data collection

#### 2.5.1 Predictors

We collected 71 candidate predictor variables from the electronic medical record system (EMRS). These variables were categorized into eight groups: (1) demographic characteristics (e.g., female age and body mass index (BMI)); (2) reproductive history (e.g., live birth history, gravidity, and parity); (3) basal hormonal profiles (e.g., FSH, luteinizing hormone (LH), estradiol (E_2_), and testosterone (T)); (4) ovarian reserve parameters (e.g., AMH and AFC); (5) endometrioma characteristics (e.g., cyst diameter and laterality, where the diameter of the endometrioma is documented as the largest measurement taken through transvaginal ultrasound before EST); (6) treatment protocols (e.g., COH protocol); (7) laboratory outcomes (e.g., number of oocytes retrieved, number of metaphaseII(MII)oocyte, and fertilization rate); and (8) embryological outcomes (e.g., number of usable embryos and number of blastocysts formed). All variables were measured before or during the IVF/ICSI treatment cycle. The linearity assumption for continuous variables was assessed using restricted cubic splines with three knots. No significant nonlinear relationships were detected; thus, continuous variables were modeled as linear terms.

#### 2.5.2 Missing data

Patients with incomplete data on the primary outcomes or key predictors were excluded from the final analysis. For the remaining candidate predictors, the missing data were minimal (<5%). A complete case analysis was performed for the model development. Missing data were assessed and found to be minimal (<5%) and missing completely at random, as confirmed by Little’s MCAR test (P>0.05). Therefore, a complete case analysis was deemed appropriate.

### 2.6 Feature selection

#### 2.6.1 Data partitioning strategy

Participants were randomly assigned to training (70%) and validation (30%) cohorts using computer-generated random sequences, with stratification by outcome to ensure balanced representation of live birth events across both datasets. A random seed was established to ensure reproducibility, and baseline characteristics were compared between cohorts to verify successful randomization.

#### 2.6.2 Three-step feature selection process

A systematic three-step variable selection methodology was employed to construct a parsimonious yet robust predictive model.

Step 1: Univariate analysis. All candidate predictors were subjected to univariate logistic regression analyses within the training cohort. Variables exhibiting associations with the primary outcome (P < 0.10) were retained for subsequent analysis. This liberal threshold was selected to prevent the premature exclusion of potentially significant predictors while maintaining statistical efficiency.

Step 2: The variables identified in Step 1 were subjected to Least Absolute Shrinkage and Selection Operator (LASSO) regression analysis, utilizing 10-fold cross-validation repeated 100 times to determine the optimal penalty parameter (λ). We adopted the one-standard-error rule (λ.1se) to select the optimal penalty parameter, which favors a more parsimonious model by choosing the largest λ value within one standard error of the minimum cross-validated error. The rationale for prioritizing a parsimonious model in this clinical context is threefold. First, models with fewer variables are more readily adopted in clinical settings, thereby enhancing their practical utility. Second, simpler models reduce overfitting and improve generalizability when applied to new populations. Third, focusing on key predictors reduces the data collection burden and costs, making it more efficient for clinical decision-making. This approach balances predictive accuracy and clinical applicability (24). LASSO regression inherently addresses multicollinearity and facilitates feature selection by reducing the coefficients of less significant variables toward zero.

Step 3: Multivariate logistic regression with stepwise selection. Variables with non-zero LASSO coefficients were included in a multivariable logistic regression analysis, utilizing backward stepwise elimination based on statistical significance (P < 0.05) to formulate the final model. The model’s assumptions were thoroughly checked, including the linearity of continuous variables (assessed through restricted cubic splines), absence of multicollinearity (variance inflation factor<5), and sufficiency of the sample size.

### 2.7 Nomogram construction and comprehensive validation

#### 2.7.1 Nomogram development

A nomogram was constructed using the final multivariable logistic regression model with the rms package in R, serving as an intuitive graphical tool for personalized CLBR prediction. It integrates all significant predictors with appropriate scaling and point allocation based on regression coefficients, allowing clinicians to compute individualized probability estimates for patient counseling and treatment planning purposes.

#### 2.7.2 Model performance evaluation

##### 2.7.2.1 Discrimination assessment

The model’s ability to distinguish between outcomes was measured using the Area Under the Receiver Operating Characteristic (ROC) curve (AUC), with 95% confidence intervals (CIs). Performance was rated as follows: 0.5, no discrimination; 0.6–0.7, poor to fair; 0.7–0.8, moderate; 0.8–0.9, good; and 0.9 or higher, excellent. Bootstrap resampling validation was performed separately on the training and validation groups to evaluate the stability and reliability of the model performance estimates. This involved taking 500 random samples with replacement from each group, with each sample being the same size as the original group. After 500 iterations, the AUC distribution was obtained, and the 95% confidence interval (CI) was calculated using the DeLong method.

##### 2.7.2.2 Calibration evaluation

The agreement between the predicted probabilities and observed frequencies of the CLBR was evaluated using calibration plots and the Hosmer-Lemeshow goodness-of-fit test. A well-calibrated model would have a calibration curve close to the 45-degree diagonal line and a P > 0.05 in the Hosmer-Lemeshow test.

##### 2.7.2.3 Clinical utility assessment

Decision curve analysis (DCA) was conducted to evaluate the net clinical benefit of the nomogram across a range of threshold probabilities, comparing the model’s performance against strategies for treating all patients or treating no patients. The analysis quantified the clinical value of the prediction model in supporting treatment decisions.

### 2.8 Sample size calculation

Sample size calculation followed the 10 events per variable (EPV) guideline for the logistic regression models (25). With an expected live birth rate of 50% and 5–8 predictor variables, 50–80 events (100–160 patients) were needed. Accounting for data exclusion and 20% follow-up loss, the target was set at 125–200 patients. According to the EPV principle, our sample size of 194 patients, which included 97 live-birth events, yielded an EPV of 19.4. This was sufficient for developing a prediction model that incorporated the five variables.

### 2.9 Statistical analysis

Continuous variables were described using the mean ± standard deviation (SD) for data with a normal distribution or median with interquartile range (IQR) for data not normally distributed, with normality checked via the Shapiro-Wilk test and visual inspection of histograms and Q-Q plots. Categorical variables were expressed as frequency and percentage. Comparisons between groups were conducted using Student’s t-test or the Mann-Whitney U test for continuous variables and the chi-square test or Fisher’s exact test for categorical variables, with effect sizes reported as Cohen’s d for continuous variables and Cramér’s V for categorical variables. We used the R software (version 4.2.2) for all statistical analyses. This included packages such as rms for regression modeling and creating nomograms, glmnet for LASSO regression, pROC for ROC analysis, and mice for multiple imputation. SPSS version 26.0 (IBM Corp., Armonk, NY, USA) was used for the supplementary analyses. All analyses employed a two-sided significance level of P < 0.05, and random seeds were used to ensure the reproducibility of the results.

## 3 Results

### 3.1 Study population, cohort allocation, and baseline characteristics

**Figure 1:**
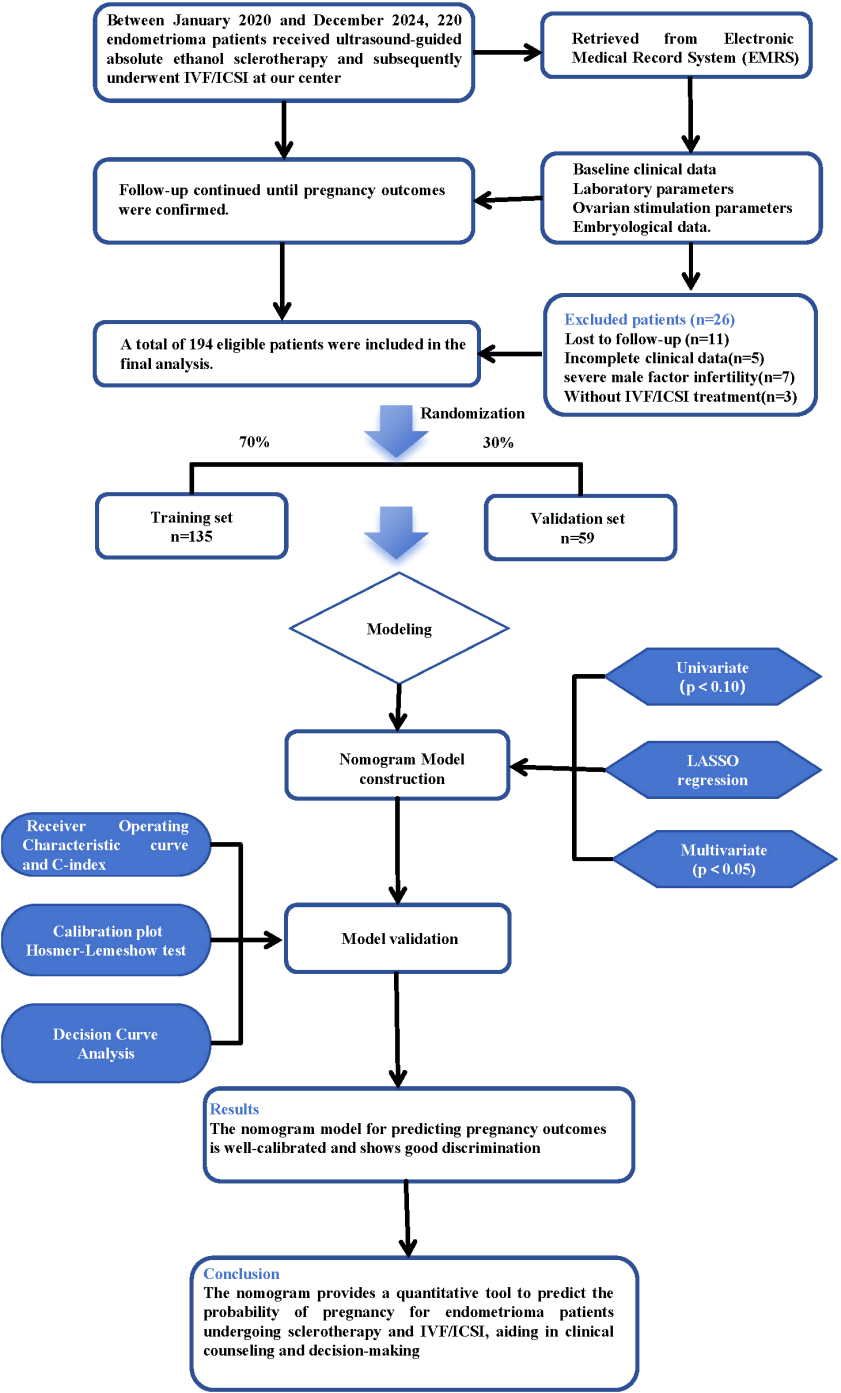
Flowchart of study population selection and cohort allocation. EMRS, Emergency Medical Response System; LASSO, Least Absolute Shrinkage and Selection Operator; IVF, *In Vitro* Fertilization; ICSI, Intracytoplasmic Sperm Injection.

This study retrospectively analyzed 220 patients with OMAs who underwent ultrasound-guided EST followed by IVF/ICSI between January 2020 and December 2024 at our institution. In accordance with the stringent inclusion and exclusion criteria, 26 patients were excluded from the study: 11 were lost to follow-up, 5 had incomplete data, 7 presented with severe male factor infertility, and 3 did not undergo IVF/ICSI. Consequently, 194 patients were deemed eligible for the final analysis (**Figure 1**). Patients were randomly split 7:3 into a training cohort (n=135, 70%) for model development and a validation cohort (n=59, 30%) for internal validation, establishing a statistical foundation. The median age of the study population was 33.0 years, with a median AMH level of 2.83 ng/mL, eight oocytes retrieved, and four usable embryos produced. Among all baseline variables, only bT (training: 28.22 ng/dL vs. validation: 22.75 ng/dL, P = 0.045) and AFC (training: 11.00 vs. validation: 9.00, P = 0.030) showed statistically significant differences. All other variables, including age, BMI, infertility features, cyst parameters, COH protocols, and embryological outcomes, were comparable (P>0.05) (**Table 1**). This confirmed successful randomization and a good baseline balance, providing a reliable dataset for the subsequent model construction.

**Table 1:** Baseline characteristics and clinical parameters of study participants.

Characteristics	All participants (n=194)	Training set (n=135)	Validation set (n=59)	p-value
Demographic Characteristics				
Female age (years)	33.00[30.00;35.75]	33.00[30.00;35.00]	33.00[31.00;36.00]	0.585
Male age (years)	34.00[32.00;37.00]	34.00[32.00;36.50]	35.00[31.00;38.00]	0.524
BMI (kg/m²)				0.732
Underweight (<18.5)	35(18.04%)	24(17.78%)	11(18.64%)	
Normal weight (18.5-23.9)	129(66.49%)	91(67.41%)	38(64.41%)	
Overweight (24.0-27.9)	24(12.37%)	17(12.59%)	7(11.86%)	
Obesity (≥28)	6(3.09%)	3(2.22%)	3(5.08%)	
Infertility Characteristics				
Duration of infertility (years)				0.939
≤ 3	114(58.76%)	79(58.52%)	35(59.32%)	
4-5	41(21.13%)	28(20.74%)	13(22.03%)	
>5	39(20.10%)	28(20.74%)	11(18.64%)	
Type of infertility				0.859
Primary	105(54.12%)	72(53.33%)	33(55.93%)	
Secondary	89(45.88%)	63(46.67%)	26(44.07%)	
Previous live birth history				0.897
Yes	40(20.62%)	27(20.00%)	13(22.03%)	
No	154(79.38%)	108(80.00%)	46(77.97%)	
Basal Hormonal Profile				
bFSH (mIU/mL)	6.44[5.29;7.65]	6.41[5.29;7.60]	6.51[5.27;7.95]	0.942
bLH (mIU/mL)	3.59[2.77;4.90]	3.59[2.71;4.94]	3.70[3.00;4.68]	0.600
bE_2_ (pg/mL)	137.67[87.89;191.14]	137.81[88.41;190.64]	137.53[88.17;199.62]	0.745
bT (ng/dL)	25.77[19.30;33.98]	28.22[19.44;34.56]	22.75[18.43;28.23]	0.045
bPRL (ng/mL)	13.05[7.89;17.79]	12.90[7.90;17.93]	14.02[8.38;17.02]	0.777
bP (ng/mL)	0.19[0.11;0.31]	0.19[0.11;0.30]	0.19[0.11;0.30]	0.695
Ovarian Reserve Parameters				
AMH (ng/mL)	2.83[1.56;4.02]	2.67[1.46;4.07]	3.06[1.95;3.99]	0.461
AFC	10.00[6.00;15.00]	11.00[6.00;16.00]	9.00[5.00;13.00]	0.030
Diminished ovarian reserve				0.270
Yes	26(13.40%)	21(15.56%)	5(8.47%)	
No	168(86.60%)	114(84.44%)	54(91.53%)	
Endometrioma parameters				
Cyst Diameter (cm)	5.54[4.83;7.46]	5.66[4.88;7.70]	5.27[4.74;6.66]	0.096
Cyst laterality				0.798
Unilateral	154(79.38%)	106(78.52%)	48(81.36%)	
Bilateral	40(20.62%)	29(21.48%)	11(18.64%)	
Cyst status				0.368
Primary	154(79.38%)	110(81.48%)	44(74.58%)	
Recurrent	40(20.62%)	25(18.52%)	15(25.42%)	
Cyst Number				0.223
Single	132(68.04%)	96(71.11%)	36(61.02%)	
Multiple	62(31.96%)	39(28.89%)	23(38.98%)	
Comorbid Conditions				
Tubal factor infertility				0.588
Yes	75(38.66%)	50(37.04%)	25(42.37%)	
No	119(61.34%)	85(62.96%)	34(57.63%)	
Adenomyosis				0.366
Yes	19(9.79%)	11(8.15%)	8(13.56%)	
No	175(90.21%)	124(91.85%)	51(86.44%)	
Treatment Protocols				
COH protocol				0.238
Ultra-long GnRH-agonist	72(37.11%)	47(34.81%)	25(42.37%)	
GnRH-antagonist	68(35.05%)	52(38.52%)	16(27.12%)	
Mild stimulation	20(10.31%)	15(11.11%)	5(8.47%)	
Long GnRH agonist	17(8.76%)	8(5.93%)	9(15.25%)	
Natural cycle	6(3.09%)	5(3.70%)	1(1.69%)	
PPOS	11(5.67%)	8(5.93%)	3(5.08%)	
Fertilization Method				0.923
Conventional IVF	148(76.29%)	104(77.04%)	44(74.58%)	
ICSI	33(17.01%)	22(16.30%)	11(18.64%)	
Rescue ICSI	13(6.70%)	9(6.67%)	4(6.78%)	
Laboratory Outcomes				
Number of oocytes retrieved	8.00[5.00;13.00]	8.00[4.50;12.00]	8.00[5.00;13.00]	0.399
Number of MII oocytes	7.00[4.00;11.00]	7.00[4.00;11.00]	8.00[5.00;11.00]	0.499
Number of usable embryos	4.00[2.00;6.00]	4.00[2.00;6.50]	4.00[3.00;5.50]	0.864

Continuous variables with a normal distribution are presented as mean ± standard deviation (SD), while those with a non-normal distribution are shown as median [interquartile range]. Categorical variables are expressed as numbers (n) and percentages (%). P-values were determined using the independent t-test for continuous variables with normal distribution, the Mann-Whitney U test for those with non-normal distribution, and the Chi-square test or Fisher’s exact test for categorical variables. AFC, Antral Follicle Count; AMH, Anti-Müllerian Hormone; BMI, Body Mass Index; bFSH, Basal Follicle-Stimulating Hormone; bLH, Basal Luteinizing Hormone; bE_2_, Basal Estradiol; bT, Basal Testosterone; bPRL, Basal Prolactin; bP, Basal Progesterone; COH, Controlled Ovarian Hyperstimulation; GnRH, Gonadotropin-Releasing Hormone; IVF, *In Vitro* Fertilization; ICSI, Intracytoplasmic Sperm Injection; PPOS, Progestin-Primed Ovarian Stimulation; MII, Metaphase II.

### 3.2 Variable screening and predictor selection

**Figure 2:**
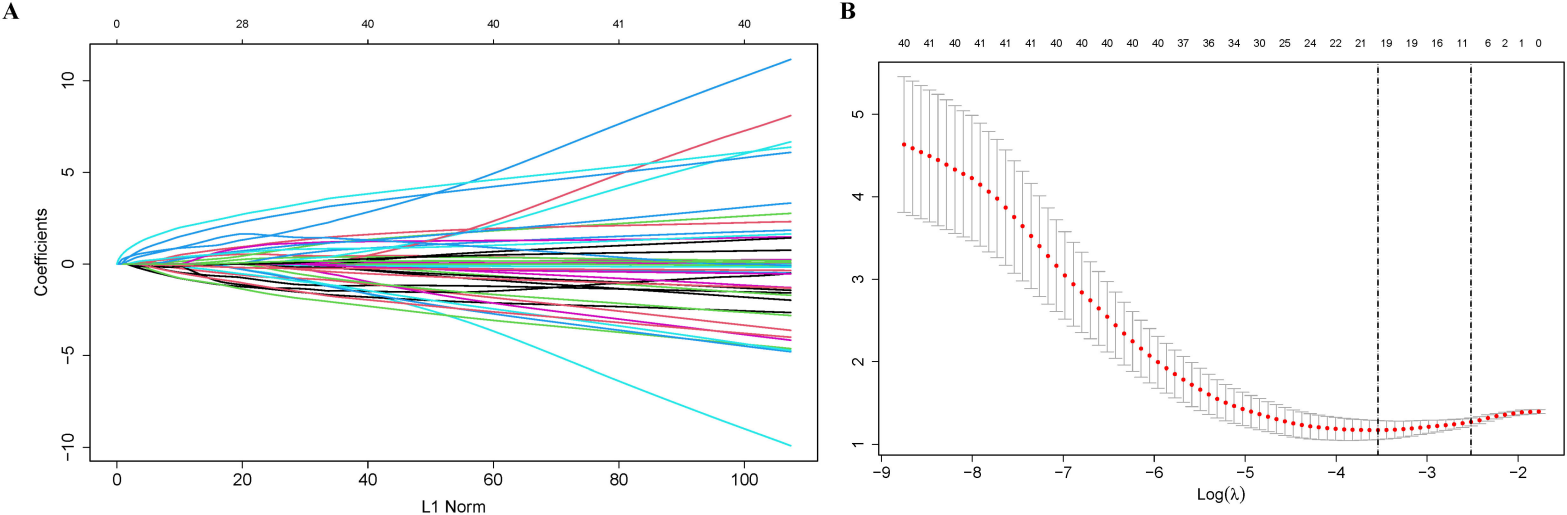
Results of the LASSO regression. **(A)** Coefficient profiles of the 26 candidate variables. **(B)** Cross-validation curve for model selection.

We conducted a univariate logistic regression analysis to evaluate the association between each candidate variable and CLBR. Seventy-one variables spanning six clinical domains were assessed. Using a liberal screening threshold of P < 0.10 to avoid prematurely discarding potentially informative predictors, 26 variables showed statistically significant associations with CLBR (**Table 2**). The complete univariate results for all 71 candidate variables are presented in **Supplementary Table S1**. From the 26 identified variables, the LASSO regression analysis, utilizing 10-fold cross-validation, determined an optimal λ value of 0.0807 (**Figures 2A, B**). Consequently, nine variables were retained: female age, male age, AFC, previous live birth history, COH protocol, number of oocytes retrieved, number of cryopreserved embryos, LH level on HCG trigger day, and cyst diameter. The nine variables were further refined using multivariate logistic regression with backward elimination. This process yielded a streamlined final model comprising four independent predictors: previous live birth history, COH protocol, total number of oocytes retrieved, and endometrioma diameter (**Table 3**).

**Table 2:** Univariate logistic regression analysis results (P < 0.10) associated with cumulative live birth rate.

Characteristics	β	SE	OR (95% CI)	Z	P-value
Female age (years)					
<30	Ref				
30-34	0.636	0.48402	1.889(0.743-5.037)	1.314	0.189
35-39	1.135	0.55187	3.111(1.077-9.504)	2.057	0.04
≥40	2.878	1.12834	17.77(2.751-354.5)	2.551	0.011
Male age (years)	0.097	0.04361	1.102(1.017-1.207)	2.23	0.026
Previous live birth history					
Yes	Ref				
No	-1.05	0.47947	0.35(0.128-0.862)	-2.19	0.029
Duration of infertility (years)					
≤3	Ref				
4-5	0.874	0.46323	2.396(0.988-6.17)	1.887	0.059
>5	0.715	0.4543	2.043(0.852-5.127)	1.573	0.116
Anti-Müllerian hormone (ng/mL)					
<1.1	Ref				
1.1-3.5	-0.721	0.51254	0.486(0.169-1.287)	-1.407	0.159
>3.5	-1.158	0.53338	0.314(0.105-0.866)	-2.171	0.03
Antral follicle count					
≤8	Ref				
8-12	0.228	0.47757	1.257(0.49-3.22)	0.478	0.632
13-17	1.065	0.48423	2.9(1.144-7.723)	2.199	0.028
≥18	1.47	0.52436	4.35(1.614-12.86)	2.804	0.005
Down-regulation					
yes	Ref				
no	0.97	0.35699	2.638(1.32-5.371)	2.718	0.007
COH protocol					
Ultra-long GnRH agonist	Ref				
GnRH antagonist	1.567	0.43578	4.79(2.079-11.55)	3.595	0
Mild stimulation	0.973	0.60822	2.647(0.816-9.146)	1.6	0.109
Long GnRH agonist	0.057	0.79088	1.059(0.198-4.874)	0.072	0.942
PPOS	0.568	0.76952	1.765(0.374-8.357)	0.738	0.46
Natural cycle	0.973	0.96202	2.647(0.401-21.64)	1.012	0.312
LH on stimulation start day (mIU/mL)	0.208	0.11036	1.231(1.014-1.552)	1.887	0.059
Progesterone on stimulation start day (ng/mL)	1.39	0.50637	4.014(1.65-11.99)	2.745	0.006
LH on HCG trigger day(mIU/mL)	0.27	0.10935	1.31(1.078-1.664)	2.472	0.013
Number of good-quality embryos	0.124	0.07002	0.883(0.765-1.009)	-1.774	0.076
Total oocytes retrieved	0.069	0.02833	0.933(0.88-0.984)	-2.438	0.015
Number of MII oocytes	0.066	0.03119	0.936(0.878-0.993)	-2.123	0.034
Number of usable embryos	0.091	0.04724	0.913(0.829-0.999)	-1.92	0.055
Number of discarded embryos	0.073	0.03743	0.929(0.86-0.997)	1.961	0.05
Frozen embryos					
<5	Ref				
≥5	-0.79	0.35467	0.454(0.224-0.904)	2.226	0.026
Frozen blastocysts					
0	Ref				
1	0.419	0.54892	0.658(0.224-1.969)	0.763	0.446
2-3	0.883	0.48852	0.414(0.156-1.07)	1.808	0.071
≥4	0.878	0.44228	0.416(0.172-0.981)	1.986	0.047
Normal fertilization rate (%)					
<60	Ref				
60-69	0.185	0.59147	0.831(0.255-2.645)	0.313	0.755
70-84	0.987	0.55369	0.373(0.121-1.082)	1.783	0.075
≥85	0.137	0.53249	0.872(0.298-2.449)	0.257	0.797
Embryo transfer stage					
Cleavage stage	Ref				
Blastocyst	0.995	0.57801	0.37(0.109-1.107)	1.722	0.085
Endometrial thickness on transfer day (mm)					
≤12	Ref				
>12	0.637	0.38401	0.529(0.246-1.118)	-1.66	0.097
Adenomyosis					
Present	Ref				
Absent	-1.407	0.802	0.245(0.036-0.998)	-1.755	0.079
Uterine fibroids					
Present					
Absent	-0.833	0.48759	0.435(0.157-1.092)	-1.709	0.087
Asthenospermia					
Present	Ref				
Absent	0.794	0.43397	2.213(0.955-5.308)	1.83	0.067
Cyst diameter (cm)					
4-5	Ref				
5-6	2.343	0.54292	10.41(3.764-32.17)	4.316	0
>6	2.708	0.5164	15(5.733-44.15)	5.244	0
Cyst number					
Single	Ref				
Multiple	0.693	0.3963	2(0.933-4.45)	1.749	0.08

Results are presented as regression coefficients (β), standard errors (SE), odds ratios (OR) with 95% confidence intervals (CI), Z statistics, and P-values. P-values < 0.05 are considered statistically significant. Ref, Reference category. AFC, Antral Follicle Count; AMH, Anti-Müllerian Hormone; CI, Confidence Interval; COH, Controlled Ovarian Hyperstimulation; GnRH, Gonadotropin-Releasing Hormone; HCG, Human Chorionic Gonadotropin; LH, Luteinizing Hormone; MII, Metaphase II; OR, Odds Ratio; PPOS, Progestin-Primed Ovarian Stimulation.

**Table 3:** Multivariate logistic regression analysis of factors associated with cumulative live birth rate.

Characteristics	B	SE	OR (95% CI)	Z	P-value
(Intercept)	-0.589	0.75449	0.554 (0.123-2.461)	-0.781	0.435
Previous live birth history	-1.272	0.62252	0.280 (0.075-0.893)	-2.042	0.041
COH protocol	1.712	0.54489	5.537 (1.969-16.95)	3.141	0.002
Ultra-long GnRH agonist	Ref				
GnRH antagonist	0.527	0.75563	1.694 (0.390-7.825)	0.698	0.485
Mild stimulation	0.873	1.0585	2.393 (0.285-19.17)	0.825	0.41
Long GnRH agonist	0.071	1.09574	1.073 (0.124-10.81)	0.065	0.948
PPOS	-0.022	0.95227	0.978 (0.149-6.808)	-0.023	0.982
Natural cycle	2.444	0.62185	11.51 (3.617-42.32)	3.93	0
Cyst diameter (cm)					
4-5	Ref				
5-6	2.444	0.62185	11.51 (3.617-42.32)	3.93	0
>6	2.969	0.61367	19.46 (6.301-71.47)	4.838	0
Number of oocytes retrieved	-0.094	0.04143	0.910 (0.835-0.984)	-2.267	0.023

Data are presented as beta coefficient (β), standard error (SE), odds ratio (OR) with 95% confidence interval (CI), Z-statistic, and P-value. P-values < 0.05 are considered statistically significant. AFC, antral follicle count; AMH, anti-Müllerian hormone; CI, confidence interval; COH, controlled ovarian hyperstimulation; GnRH, gonadotropin-releasing hormone; HCG, human chorionic gonadotropin; LH, luteinizing hormone; MII, metaphase II; OR, odds ratio; PPOS, progestin-primed ovarian stimulation; Ref, reference category; SE, standard error.

### 3.3 Nomogram model development

**Figure 3:**
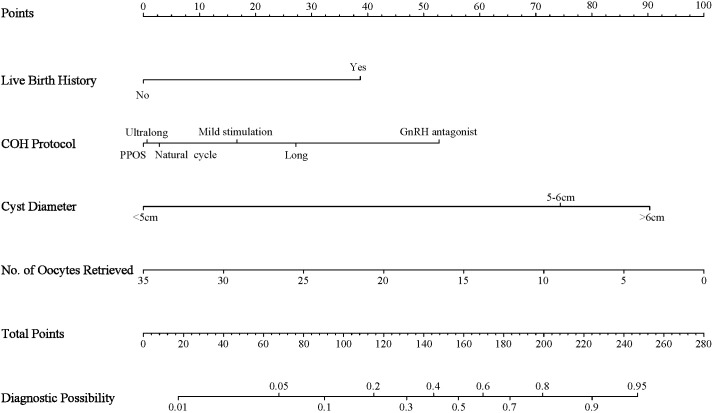
Nomogram for predicting cumulative live birth rate in women with ovarian endometriomas after ethanol sclerotherapy and subsequent IVF/ICSI. To use the nomogram, locate the patient’s value for each predictor on the corresponding axis, draw a vertical line to the ‘Points’ axis to determine the points, sum the points for all predictors, and locate the total points on the ‘Total Points’ axis. Draw a vertical line down to the ‘Diagnostic Possibility’ axis to obtain the predicted CLBR. COH, controlled ovarian hyperstimulation; PPOS, progestin-primed ovarian stimulation; GnRH, Gonadotropin−Releasing Hormone.

We constructed a nomogram to predict the CLBR in women with OMAs after EST and IVF/ICSI treatment. The nomogram included previous live birth history, COH protocol, cyst diameter, and number of oocytes retrieved (**Figure 3**). To calculate individualized CLBR prediction probabilities using the nomogram, first, find values for the five predictors and read their scores on the “Points” scale. Then, sum these scores to get “Total Points.” Finally, the total points were projected downward to the “Diagnostic Possibility” scale to determine the predicted CLBR probability. Model calibration showed a clear link between total points and the predicted CLBR probability. A total score of 90 corresponded to a 10% CLBR, 115 to 20%, 131 to 30%, 145 to 40%, 157 to 50%, 170 to 60%, 183 to 70%, 199 to 80%, and 224 to 90%. This probability gradient helps doctors predict patient outcomes, provide advice, and set realistic treatment goals and decisions.

### 3.4 Discrimination assessment

**Figure 4:**
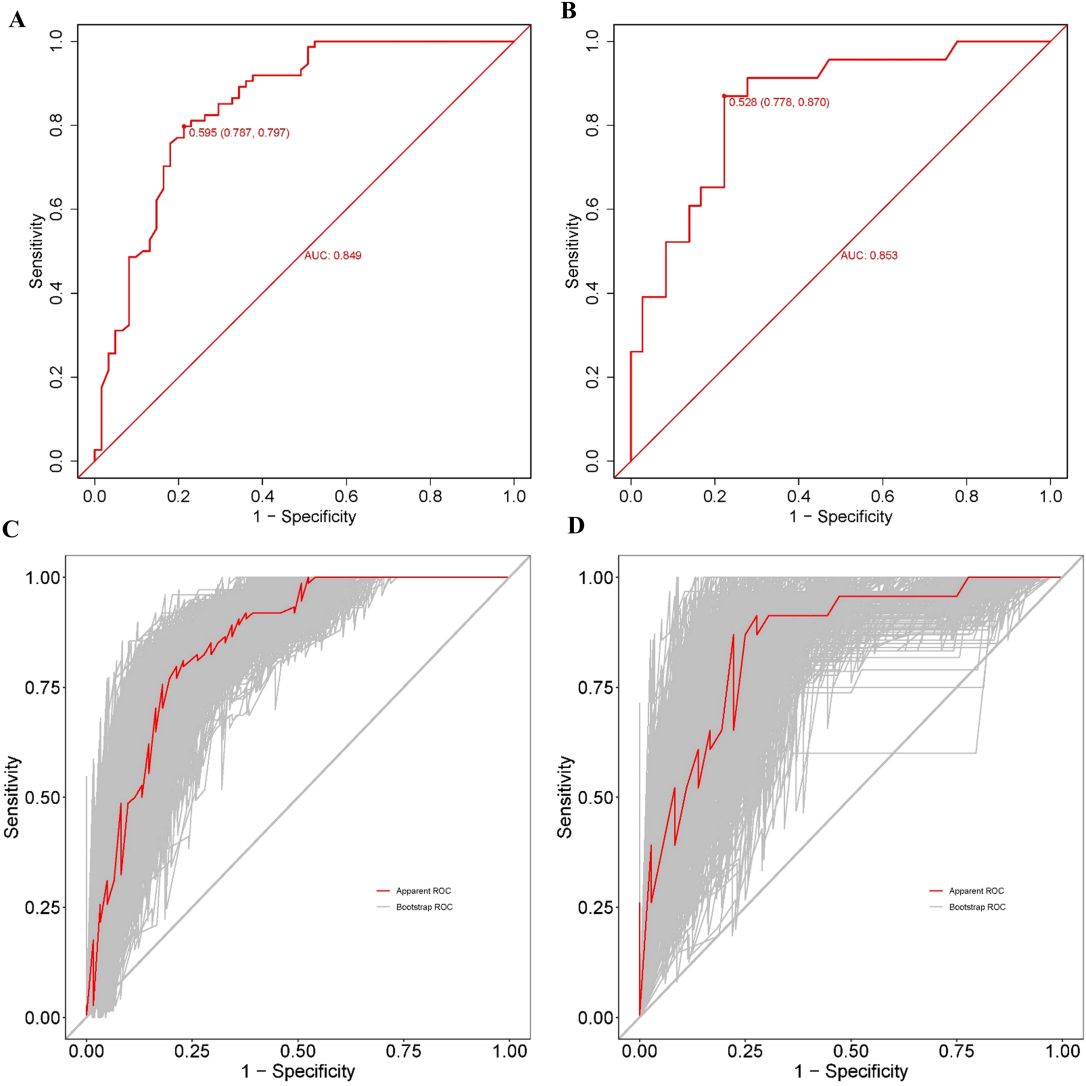
Receiver operating characteristic (ROC) curves of the nomogram for predicting the cumulative live birth rate. **(A)** ROC curve of the training set. **(B)** ROC curve for the validation set. **(C)** ROC curve of the training set after 500 bootstrap resampling. **(D)** ROC curve of the validation set after 500 bootstrap resampling. The red line represents the apparent ROC, and the gray lines represent the ROC curves generated from 500 bootstrap samples. ROC, receiver operating characteristic; AUC, area under the ROC curve.

The discriminatory capacity of the nomogram was assessed using ROC curve analysis in both datasets to differentiate between patients who achieved and did not achieve cumulative live birth. The nomogram showed good discrimination, with an AUC of 0.849 (95% CI: 0.782-0.912) in the training dataset (**Figure 4A**) and 0.853 (95% CI: 0.754-0.952) in the validation dataset (**Figure 4B**). The AUC in the validation set was higher than that in the training set, suggesting minimal overfitting and good model generalizability. Overlapping CIs indicate the model’s stable discriminative ability. The DeLong test indicated no statistically significant difference between the AUCs of the training and validation sets (P = 0.9527), suggesting consistent discriminative performance across the datasets. Bootstrap resampling validation with 500 iterations was performed for both cohorts. The Bootstrap-validated AUC was 0.877 (95% CI: 0.817-0.937) in the training cohort and 0.879 (95% CI: 0.792-0.967) in the validation cohort, as shown in **Figures 4C, D**. Bootstrap CIs aligned with the original ROC analysis confirmed the robustness of the model discrimination. The Bootstrap AUC in the validation cohort exceeded that in the training cohort, demonstrating strong generalizability without overfitting.

### 3.5 Nomogram calibration analysis

**Figure 5:**
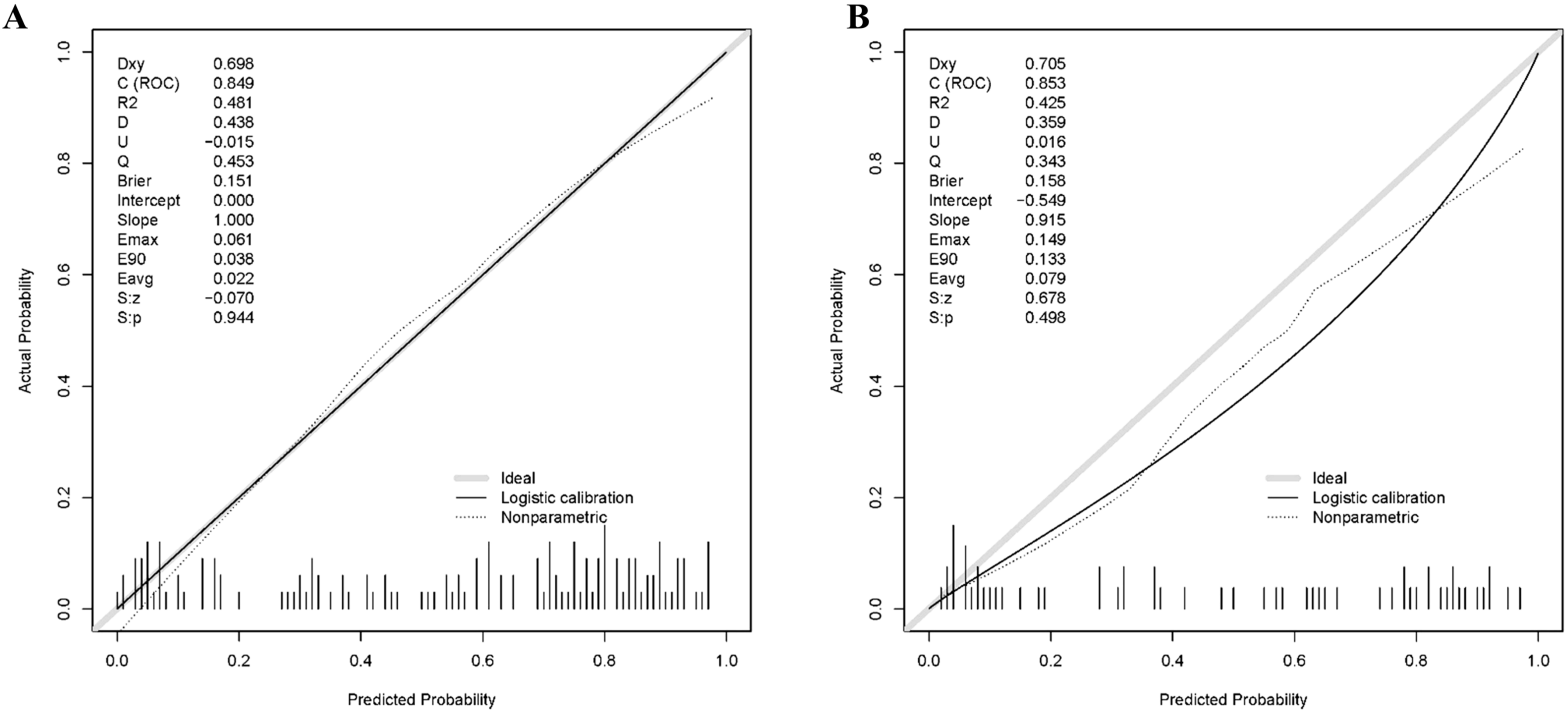
Calibration analysis of the nomogram for predicting cumulative live birth rate. **(A)** Calibration curve for the training set. **(B)** Calibration curve for the validation set.

Calibration analysis was performed to evaluate the agreement between the predicted probabilities and the observed outcomes. Calibration plots were constructed to visually assess the relationship between the predicted and actual CLBR. In the training set, the model demonstrated good calibration, with the calibration curve generally aligned with the ideal line. The key calibration metrics included a Brier score of 0.151, an intercept of 0.000, and a slope of 1.000, indicating accurate probability estimates without any systematic bias. Furthermore, the Hosmer-Lemeshow goodness-of-fit test yielded a non-significant result (P = 0.415), supporting the conclusion that there was no statistically significant difference between the predicted and observed CLBR. In the validation set, the model maintained a good calibration (**Figure 5B**). The Brier score was 0.158, which is comparable to that of the training set and indicates a low prediction error. The calibration intercept was -0.549 with a slope of 0.915, indicating a slight tendency to overestimate risk at lower probabilities and underestimate it at higher ones; nonetheless, the overall performance remained robust. Crucially, the Hosmer-Lemeshow test was also non-significant in the validation cohort (P = 0.083), confirming that the model’s predictions were well-calibrated and reliable for external data.

### 3.6 Decision curve analysis

**Figure 6:**
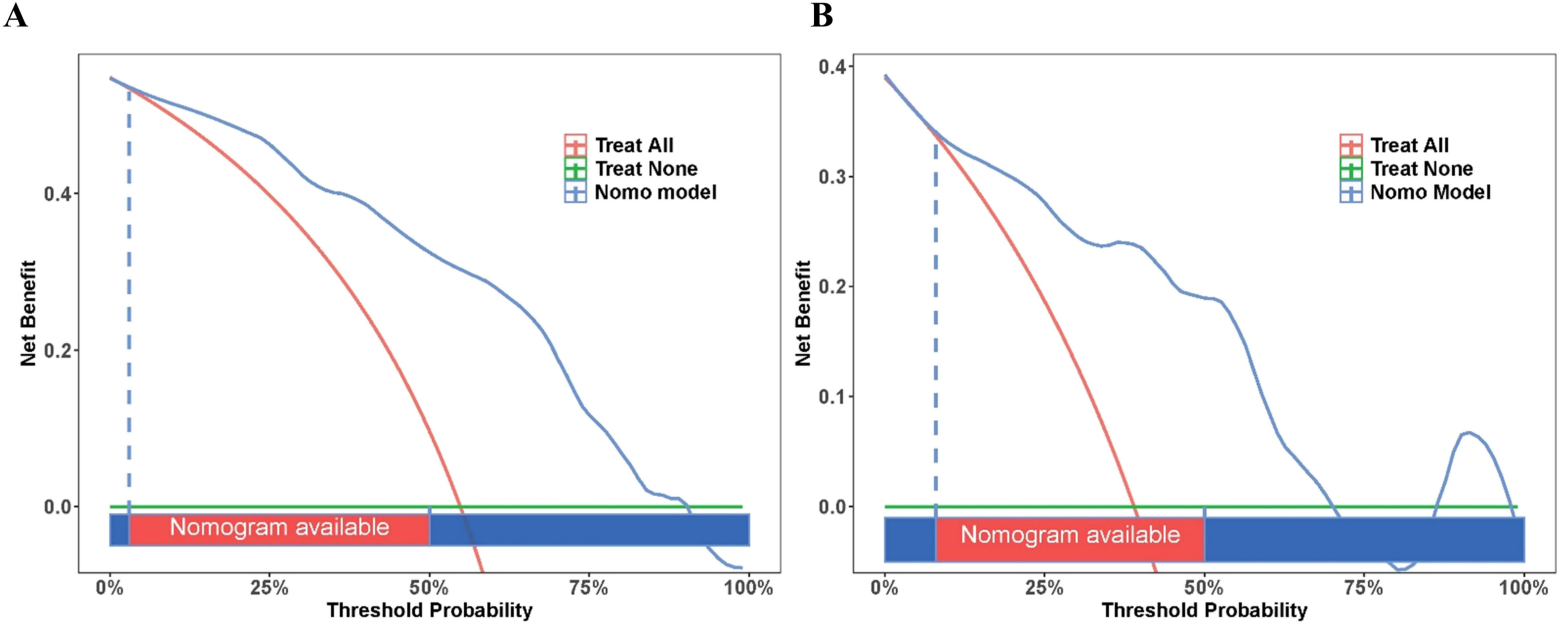
Decision curve analysis for the nomogram predicting cumulative live birth rate. **(A)** Decision curve analysis for training set. **(B)** Decision curve analysis for validation set. The blue line represents the nomogram model, the red line represents the “treat all” strategy, and the green line represents the “treat none” strategy. The x-axis shows the threshold probability, and the y-axis shows the net benefit. The shaded area at the bottom indicates the range in which the nomogram is recommended for clinical use.

DCA was conducted to evaluate the clinical utility and net benefit of using the nomogram for decision-making in both the training and validation sets (**Figure 6**). DCA determines the range of threshold probabilities at which the model provides a greater net benefit than the default strategies of treating all or none of the patients. In the training set (**Figure 6A**), the nomogram demonstrated a significant net benefit across a wide range of threshold probabilities, from approximately 5% to 90%. This indicates that if a clinician’s threshold for intervention lies within this range, using the nomogram to guide decisions is superior to “treat all” and “treat none” patients. The model achieved a peak net benefit of approximately 0.48 at a 10% threshold. Even at more moderate thresholds, the benefit remained substantial; for instance, at a threshold probability of 25%, the net benefit was approximately 0.40, and at 50%, it was approximately 0.30. This means that at a 25% threshold, using the model is equivalent to a strategy that correctly identifies 40 additional CLBR cases per 100 patients without increasing unnecessary interventions compared with the default strategies. In the validation set (**Figure 6B**), the clinical value of the model was confirmed. The nomogram provided a positive net benefit for threshold probabilities ranging from approximately 5%–85%. The net benefit curve for the nomogram was consistently higher than that of both the “treat all” and “treat none” strategies within this range. The peak net benefit in the validation cohort was approximately 0.38, observed at a low threshold of approximately 5-10%. At the 25% threshold, the net benefit was approximately 0.25, and at the 40% threshold, it was approximately 0.20. These results robustly demonstrate that the nomogram is not only statistically accurate but also clinically practical, offering tangible benefits for guiding personalized treatment decisions in women with OMAs undergoing IVF/ICSI.

## 4 Discussion

### 4.1 Principal findings and clinical significance

In this study, we developed and validated a comprehensive nomogram to predict CLBR in women with OMAs undergoing EST and IVF/ICSI. Our model incorporated four clinical variables: previous live birth history, cyst diameter, COH protocol, and number of oocytes retrieved, showing good discriminative ability with AUCs of 0.849 and 0.853 in the training and validation sets, respectively. It demonstrated good calibration and clinical utility for individualized fertility counseling in the study. Bootstrap resampling validation (500 iterations) further confirmed the robustness of the model, with Bootstrap AUCs of 0.877 and 0.879 in the training and validation sets, respectively. The Bootstrap CIs were consistent with the original analysis, demonstrating reliable model performance estimates. The development of this nomogram addresses a critical gap, as women with OMAs undergoing ovary-sparing EST represent a unique patient subset. Lavadia et al. (9) confirmed that EST is a viable treatment option that preserves the ovarian reserve and improves IVF/ICSI outcomes compared to cystectomy. Olawade et al. (26) demonstrated that AI-driven personalized treatment protocols for IVF could optimize ovarian stimulation and improve clinical outcomes using individualized approaches. Our model builds on this by providing a tailored prognostic tool that quantifies individual success probabilities, moving beyond generalized treatment recommendations to facilitate precise, patient-centered, and clinical decision-making. Moreover, our model can be integrated into clinical workflows at various decision points, ranging from the initial consultation to treatment monitoring, thereby facilitating the advancement of precision in reproductive medicine.

### 4.2 Interpretation of key predictors

#### 4.2.1 Endometrioma diameter: balancing disease burden and ovarian function

The inclusion of endometrioma diameter as a significant negative predictor highlights the complex interplay between cyst size, disease burden, and reproductive outcomes. Our findings are corroborated by evidence demonstrating that endometriomas measuring ≥3 cm are associated with significantly lower live birth rates (28.6% vs. 70.0%; P = 0.004) than smaller cysts, even when ovarian reserve parameters are comparable (27). This size-dependent effect occurs through multiple mechanisms, including inflammatory infiltration, pro-inflammatory cytokine production, and mechanical distortion of the ovarian architecture, which impairs the follicular microenvironment (28, 29). Although an overall reduction in live birth rates with EMs was not observed in some studies, larger cysts have been shown to exert more pronounced local effects (30). Therefore, EST represents a minimally invasive alternative to surgery, which has been highlighted as a risk for further compromise of the ovarian reserve (31).

#### 4.2.2 Number of oocytes retrieved: quantifying ovarian response and reproductive potential

The positive correlation between the number of oocytes retrieved and CLBR is among the most well-established findings in reproductive medicine, highlighting the critical role of ovarian response in determining treatment success. A pivotal study by Becker et al. (32) provided a comprehensive analysis of one-year CLBRs, showing that CLBR surpassed 60% when more than 15 oocytes were retrieved and exceeded 70% with over 20 oocytes, although this relationship was influenced by maternal age. This aligns with the finding that 8–14 oocytes may be optimal for fresh transfer cycles, whereas a larger yield confers a cumulative benefit (33). Furthermore, a study by Chai et al. (34) in women with an expected poor ovarian response according to the Bologna criteria showed that those who had more than three oocytes retrieved achieved significantly higher CLBR than those with ≤3 oocytes (44.0% vs. 18.6%, P = 0.006), highlighting the critical role of oocyte yield even in challenging patient groups such as those with endometriosis.

#### 4.2.3 Controlled ovarian hyperstimulation protocol: tailoring treatment to disease characteristics

The selection of a COH protocol in women with EMs is critical, extending beyond ovarian response to encompass the interplay between hormonal stimulation, inflammation, and reproductive outcomes. Our model’s identification of the COH protocol as a significant predictor shows it is a key therapeutic intervention. However, the optimal protocol remains debated, with evidence suggesting that a uniform approach is inadequate for this heterogeneous patient population. The GnRH agonist long protocol is favored as pituitary desensitization can reduce pelvic inflammation in EMs. This pre-stimulation period may improve endometrial receptivity and follicular environment. Anti-inflammatory protocols may benefit severe EMs or cases with elevated inflammatory markers (35). A retrospective study by Kolanska et al. (36) provided evidence supporting this, demonstrating that a GnRH agonist protocol resulted in significantly higher live birth rates after fresh embryo transfer than an antagonist protocol. They suggested the antagonist protocol might impair endometrial receptivity in fresh cycles, aligning with endometriosis’s known effects on the endometrium (37). However, these potential benefits must be weighed against the longer treatment duration and higher gonadotropin requirement. In contrast, the GnRH antagonist protocol offers a more flexible, shorter, and patient-friendly regimen with a significantly lower risk of Ovarian Hyperstimulation Syndrome (OHSS). A more recent and comprehensive systematic review by Kuan et al. (38) found that both GnRH agonist and antagonist protocols generally yielded similar clinical pregnancy and live birth rates. This suggests that with the now-common practice of a ‘freeze-all’ strategy, any potential negative impact of the antagonist protocol on the endometrium in a fresh cycle can be effectively bypassed. The review noted that the GnRH agonist protocol might yield a higher total number of oocytes, which could translate to a higher cumulative pregnancy rate, reinforcing the importance of oocyte yield in this patient group (38). Recently, the PPOS protocol has emerged as a viable all-oral alternative that effectively prevents premature LH surges and enhances patient comfort. Its role in endometriosis, particularly in patients with diminished ovarian reserve after surgery, is an area of active investigation. A retrospective study by Yang et al. (39) compared PPOS with ultra-long GnRH agonist and antagonist protocols in patients with endometriomas. The study found that while PPOS was associated with a lower live birth rate than the ultra-long GnRH agonist protocol in the first embryo transfer cycle (OR 2.5, 95% CI 1.1-5.7), its outcomes were comparable to those of the GnRH antagonist protocol. Another study by Li et al. (40) focusing on patients with DOR after OMA surgery found no significant difference in the CLBR among GnRH antagonist, PPOS, and mild-stimulation protocols, although it concluded that both antagonist and PPOS protocols were superior to mild-stimulation. Murria et al. (41) found that PPOS cycles might produce lower-quality blastocysts than agonist and antagonist protocols do. Evidence shows that PPOS and GnRH antagonist protocols are effective, although PPOS may offer advantages, such as improved cycle flexibility and reduced inflammation (42). This finding highlights the ongoing debate and reinforces the fact that no single protocol is universally superior. This finding highlights the ongoing debate and reinforces the fact that no single protocol is universally superior to the others. Thus, the selection process should be based on a detailed analysis of the patient’s specific clinical profile, which includes ovarian reserve, past treatment responses, and distinct inflammatory and hormonal conditions linked to endometriosis, rather than adopting a generalized approach.

#### 4.2.4 Previous live birth history: the paradox of secondary infertility in progressive endometriosis

A counterintuitive yet clinically insightful finding from our model is that a history of previous live births acts as a negative predictor of CLBR. This challenges the conventional belief that parous women conceive more easily and highlights the nature of EMs as a chronic, progressive disease. We focus our interpretation of this phenomenon on three core concepts: “secondary infertility,” “disease progression,” and “interpregnancy interval” interpregnancy interval. First, EMs is not a static condition; its pathophysiological processes evolve over time. Even if a woman has previously achieved a successful pregnancy naturally or with simple assistance, the underlying EMs lesions can continue to progress postpartum. Inflammation, oxidative stress, and hormonal resistance persistently erode reproductive potential. A review showed that inflammation and oxidative stress are key mechanisms by which EMs impairs fertility, affecting oocyte quality, sperm, and embryo viability (43). When these patients seek fertility treatment years later, they face a more hostile internal environment than during their first pregnancy. IVF/ICSI requirement may indicate selection bias of advanced disease stages. The interpregnancy interval is crucial, with a retrospective ART study showing decreased pregnancy and live birth rates for intervals ≥24 months compared to 12–18 months (44). For patients with EMs, this time window is critical as it corresponds to disease progression. Although secondary infertility has a better prognosis in the general infertile population (45), the progressive nature of EMs makes it a special case. For parous patients with EMs, prior live births likely occurred earlier in disease progression, while current infertility reflects cumulative damage. A national cohort study showed women with EMs have lower first-birth and fertility rates than the general population, further supporting the persistent negative impact of EMs on fertility (46). The impact of EMs on fertility varies by age, with a prospective cohort study showing stronger infertility risk association in women under 35 (47). For women diagnosed with EMs at a younger age, cumulative fertility damage may be more pronounced over time. A history of previous live births should not be viewed as a good prognostic indicator in EMs patients, but rather as a timestamp indicating longer disease development. This emphasizes the need for comprehensive evaluation of parous patients with endometriosis and secondary infertility. Clinicians should not be reassured by previous successful pregnancy but remain vigilant about disease progression impacts. The negative link between previous births and CLBR needs cautious interpretation due to potential selection bias. Women with a history of live birth who subsequently seek IVF treatment may represent a subgroup with more advanced or progressive disease, as they were able to conceive naturally or with less invasive treatments in the past but now require IVF due to the disease progression.

### 4.3 Clinical implications and applications

This nomogram serves as a practical tool for translating complex statistical data into individualized clinical guidance, fostering shared decision-making for women with OMAs undergoing IVF/ICSI after EST. Its primary utility lies in pre-treatment counseling, where it provides a quantitative estimate of the CLBR to manage patient expectations. Based on our DCA results, a threshold probability of 30% was a reasonable benchmark. For patients with a predicted CLBR above this threshold, clinicians can offer reassurance and encourage proceeding with IVF/ICSI treatment. Conversely, for those with a prognosis below 30%, the model facilitates crucial discussions on alternative strategies. This may include optimizing the COH protocol, considering a repeat EST for larger cysts, or realistically counseling on the potential need for multiple retrieval cycles. It is imperative to underscore that this nomogram was designed as a decision-support tool to augment, not supplant, clinical judgment. The predictions provided by the model should be integrated with the clinician’s experience, the patient’s individual circumstances, and shared decision-making. It serves as an objective reference to facilitate more personalized counseling and expectation management, helping both clinicians and patients to better understand the likelihood of success before embarking on the IVF/ICSI journey.

### 4.4 Study strength and limitations

This study had several notable strengths that enhanced the validity and clinical applicability of our findings. To our knowledge, this is the first study to develop and validate a prediction model specifically for the CLBR in women with OMAs following EST and subsequent IVF/ICSI treatment, addressing a critical gap in reproductive medicine. Second, we employed a rigorous statistical methodology adhering to the TRIPOD guidelines, including a comprehensive three-stage variable selection process (univariate regression, LASSO regression, and multivariate logistic regression) to minimize overfitting and ensure the parsimony of the model. Third, Bootstrap resampling validation with 500 iterations provided a robust assessment of model stability and reproducibility, confirming the reliability of our predictions. Fourth, we used CLBR as the primary outcome, which is more clinically meaningful than the single-cycle pregnancy rate, as it captures the full reproductive potential of all embryos from a single oocyte retrieval cycle. Finally, the model demonstrated consistent performance across both the training and validation cohorts, with good discrimination (AUC 0.849-0.853), acceptable calibration, and favorable clinical utility, as evidenced by decision curve analysis, supporting its potential for clinical implementation.

This study has some limitations that should be considered when interpreting its findings. First, the retrospective, single-center design may limit its generalizability. Although our sample size of 194 patients was adequate, larger multicenter cohorts are required for robustness. Second, our model excluded variables such as genetic polymorphisms, inflammatory biomarkers, and surgical history due to data constraints. Future studies should include these factors to improve performance. Third, the finding that a history of previous live birth is a negative predictor requires cautious interpretation, possibly reflecting endometriosis progression, selection bias, or unmeasured confounders such as the time interval since the last live birth. External validation is essential for confirming this association. Another limitation of this study is the statistically significant difference in two key ovarian reserve markers—basal testosterone and AFC—between the training and validation sets, despite random allocation. This baseline imbalance could potentially challenge the assessment of the model’s generalizability. Ideally, methods such as Propensity Score Matching or rigorous covariate adjustment should be employed to eliminate such confounding bias. Due to the retrospective nature and limited sample size of our study, we did not implement these more complex statistical corrections. Although our model demonstrated good predictive performance on the validation set despite the baseline differences, which to some extent proves its robustness, we must acknowledge that this methodological shortcoming may limit the direct applicability of our findings. Future research, particularly in larger prospective cohorts, should prioritize ensuring comparability between groups through strict matching or stratified designs to obtain more reliable validation results.

### 4.5 Future directions

While this study establishes a foundation for individualized CLBR prediction in women with OMAs after EST and IVF/ICSI, several directions warrant further investigation. First, external validation in independent multicenter cohorts across diverse geographic regions and clinical settings is essential to confirm the generalizability and robustness of the nomogram. Second, prospective studies are needed to evaluate the model’s performance in real-world clinical practice and assess its impact on clinical decision-making, patient satisfaction, and reproductive outcomes. Third, the incorporation of emerging biomarkers (e.g., AMH kinetics, inflammatory markers, and genetic polymorphisms) and advanced analytical techniques (e.g., machine learning algorithms and artificial intelligence) may further enhance predictive accuracy and enable dynamic risk stratification. Finally, extending this prediction framework to other endometrioma management strategies (e.g., laparoscopic cystectomy and expectant management) and investigating the cost-effectiveness of nomogram-guided treatment selection represent important areas for future research to optimize fertility care in women with OMAs.

## 5 Conclusion

We developed and validated the first nomogram to predict the CLBR in women undergoing IVF/ICSI after EST for OMAs. The model incorporates four clinical variables (previous live birth history, cyst diameter, COH protocol, and number of oocytes retrieved) and demonstrates good discrimination (AUC 0.849-0.853), acceptable calibration, and favorable clinical utility. Notably, a history of previous live birth was identified as a negative predictor of CLBR, providing new insights into reproductive outcomes in this population. This nomogram acts as a useful resource for personalized fertility advice and treatment planning, facilitating collaborative decision-making in clinical settings.

## Data Availability

The original contributions presented in the study are included in the article/**Supplementary Material**. Further inquiries can be directed to the corresponding authors.
