# Supplementary material for: Development and validation of a clinical nomogram for predicting cumulative live birth rate in ovarian endometrioma patients undergoing ethanol sclerotherapy and in vitro fertilization/intracytoplasmic sperm injection
Source: Front Endocrinol (Lausanne). 2026 Feb 6;17:1738786. doi: 10.3389/fendo.2026.1738786 (PMC12920196; doi:10.3389/fendo.2026.1738786)
Supplement: Supplementary file 1 [file Table1.docx]

Supplementary Material

# Supplementary Tables

| **Characteristics** | **β** | **SE** | **OR (95% CI)** | **Z** | **P-value** |
| --- | --- | --- | --- | --- | --- |
| **Demographic Characteristics** |  |  |  |  |  |
| Female age (years) |  |  |  |  |  |
| <30 | Ref |  |  |  |  |
| 30-34 | 0.636 | 0.48402 | 1.889(0.743-5.037) | 1.314 | 0.189 |
| 35-39 | 1.135 | 0.55187 | 3.111(1.077-9.504) | 2.057 | 0.04 |
| ≥40 | 2.878 | 1.12834 | 17.77(2.751-354.5) | 2.551 | 0.011 |
| Male age (years) | 0.097 | 0.04361 | 1.102(1.017-1.207) | 2.23 | 0.026 |
| BMI (kg/m²) |  |  |  |  |  |
| Underweight (＜18.5) | Ref |  |  |  |  |
| Normal weight (18.5-23.9) | 0.366 | 0.46068 | 1.441(0.584-3.612) | 0.793 | 0.428 |
| Overweight (24.0-27.9) | 0.773 | 0.65223 | 2.167(0.616-8.156) | 1.185 | 0.236 |
| Obesity (≥28.0) | 0.86 | 1.29145 | 2.364(0.2-55.00) | 0.666 | 0.505 |
| **Reproductive History** |  |  |  |  |  |
| Live birth history |  |  |  |  |  |
| yes | Ref |  |  |  |  |
| no | -1.05 | 0.47947 | 0.35(0.128-0.862) | -2.19 | 0.029 |
| Duration of infertility (years) |  |  |  |  |  |
| ≤3 | Ref |  |  |  |  |
| 4-5 | 0.874 | 0.46323 | 2.396(0.988-6.17) | 1.887 | 0.059 |
| >5 | 0.715 | 0.4543 | 2.043(0.852-5.127) | 1.573 | 0.116 |
| Type of infertility |  |  |  |  |  |
| Primary |  |  |  |  |  |
| Secondary | 0.297 | 0.34798 | 1.346(0.682-2.677) | 0.854 | 0.393 |
| **Basal Hormonal Profile** |  |  |  |  |  |
| bFSH (mIU/mL) | 0.011 | 0.05751 | 1.011(0.903-1.137) | 0.184 | 0.854 |
| bLH (mIU/mL) | 0.001 | 0.0024 | 1.001(1-NA) | 0.356 | 0.722 |
| bE2 (pg/mL) | 0.017 | 0.01365 | 1.017(0.992-1.048) | 1.212 | 0.226 |
| bT (ng/dL) | 0 | 0.00016 | 1(1-1) | 0.127 | 0.899 |
| bP | -0.005 | 0.00725 | 0.995(0.969-1) | -0.654 | 0.513 |
| bPRL (ng/mL) | 0.102 | 0.18532 | 1.107(0.811-2.111) | 0.55 | 0.582 |
| **Ovarian Reserve parameters** |  |  |  |  |  |
| Anti-Müllerian hormone (ng/mL) |  |  |  |  |  |
| <1.1 | Ref |  |  |  |  |
| 1.1-3.5 | -0.721 | 0.51254 | 0.486(0.169-1.287) | -1.407 | 0.159 |
| >3.5 | -1.158 | 0.53338 | 0.314(0.105-0.866) | -2.171 | 0.03 |
| Antral follicle count |  |  |  |  |  |
| ≤8 | Ref |  |  |  |  |
| 8-12 | 0.228 | 0.47757 | 1.257(0.49-3.22) | 0.478 | 0.632 |
| 13-17 | 1.065 | 0.48423 | 2.9(1.144-7.723) | 2.199 | 0.028 |
| ≥18 | 1.47 | 0.52436 | 4.35(1.614-12.86) | 2.804 | 0.005 |
| Diminished ovarian reserve |  |  |  |  |  |
| Present | Ref |  |  |  |  |
| Absent | -0.588 | 0.49947 | 0.556(0.198-1.44) | -1.177 | 0.239 |
| Ovulatory disorder |  |  |  |  |  |
| Present | Ref |  |  |  |  |
| Absent | 0.202 | 0.83546 | 1.224(0.219-6.83) | 0.242 | 0.809 |
| **Treatment Protocol** |  |  |  |  |  |
| Down-regulation |  |  |  |  |  |
| yes | Ref |  |  |  |  |
| no | 0.97 | 0.35699 | 2.638(1.32-5.371) | 2.718 | 0.007 |
| COH protocol |  |  |  |  |  |
| Ultra-long GnRH agonist | Ref |  |  |  |  |
| GnRH antagonist | 1.567 | 0.43578 | 4.79(2.079-11.55) | 3.595 | 0 |
| Mild stimulation | 0.973 | 0.60822 | 2.647(0.816-9.146) | 1.6 | 0.109 |
| Long GnRH agonist | 0.057 | 0.79088 | 1.059(0.198-4.874) | 0.072 | 0.942 |
| PPOS | 0.568 | 0.76952 | 1.765(0.374-8.357) | 0.738 | 0.46 |
| Natural cycle | 0.973 | 0.96202 | 2.647(0.401-21.64) | 1.012 | 0.312 |
| Fertilization Method |  |  |  |  |  |
| Conventional IVF | Ref |  |  |  |  |
| ICSI | 0.491 | 0.49869 | 1.634(0.633-4.589) | 0.985 | 0.325 |
| Rescue ICSI | -17.837 | 1318.72679 | 0(NA-2.128) | -0.014 | 0.989 |
| **Ovarian Stimulation Parameters** |  |  |  |  |  |
| Gonadotropin starting dose (IU) | 0.002 | 0.0025 | 1.002(0.997-1.007) | 0.706 | 0.48 |
| Total gonadotropin dose (IU) | 0 | 0.00022 | 1(1-1.001) | 0.618 | 0.537 |
| Duration of gonadotropin stimulation (days) | -0.07 | 0.07279 | 0.933(0.805-1.074) | -0.955 | 0.34 |
| **Hormonal levels on stimulation start day** |  |  |  |  |  |
| Estradiol on stimulation start day (pg/mL) | 0 | 0.00028 | 1(0.999-1) | -0.441 | 0.659 |
| FSH on stimulation start day (mIU/mL) | 0.095 | 0.06574 | 1.1(0.974-1.262) | 1.451 | 0.147 |
| LH on stimulation start day (mIU/mL) | 0.208 | 0.11036 | 1.231(1.014-1.552) | 1.887 | 0.059 |
| Progesterone on stimulation start day (ng/mL) | 1.39 | 0.50637 | 4.014(1.65-11.99) | 2.745 | 0.006 |
| **Hormonal levels on HCG trigger day** |  |  |  |  |  |
| Estradiol on HCG trigger day (pg/mL) | 0 | 0.00002 | 1(1-1) | -1.629 | 0.103 |
| Progesterone on HCG trigger day (ng/mL) | -0.156 | 0.13601 | 0.855(0.649-1.113) | -1.148 | 0.251 |
| LH on HCG trigger day(mIU/mL) | 0.27 | 0.10935 | 1.31(1.078-1.664) | 2.472 | 0.013 |
| **Laboratory Outcomes** |  |  |  |  |  |
| Number of good-quality embryos | -0.124 | 0.07002 | 0.883(0.765-1.009) | -1.774 | 0.076 |
| Total oocytes retrieved | -0.069 | 0.02833 | 0.933(0.88-0.984) | -2.438 | 0.015 |
| Number of MII oocytes | -0.066 | 0.03119 | 0.936(0.878-0.993) | -2.123 | 0.034 |
| Number of usable embryos | -0.091 | 0.04724 | 0.913(0.829-0.999) | -1.92 | 0.055 |
| Number of discarded embryos | -0.073 | 0.03743 | 0.929(0.86-0.997) | -1.961 | 0.05 |
| **Embryo cryopreservation** |  |  |  |  |  |
| Frozen embryos |  |  |  |  |  |
| <5 | Ref |  |  |  |  |
| ≥5 | -0.79 | 0.35467 | 0.454(0.224-0.904) | -2.226 | 0.026 |
| Number of cryopreserved embryos | -0.1 | 0.06086 | 0.905(0.798-1.016) | -1.646 | 0.1 |
| Number of cryopreserved cleavage-stage embryos | -0.075 | 0.15205 | 0.928(0.686-1.251) | -0.492 | 0.623 |
| Number of cryopreserved blastocysts | -0.109 | 0.06648 | 0.897(0.783-1.018) | -1.64 | 0.101 |
| Frozen blastocysts |  |  |  |  |  |
| 0 | Ref |  |  |  |  |
| 1 | -0.419 | 0.54892 | 0.658(0.224-1.969) | -0.763 | 0.446 |
| 2-3 | -0.883 | 0.48852 | 0.414(0.156-1.07) | -1.808 | 0.071 |
| ≥4 | -0.878 | 0.44228 | 0.416(0.172-0.981) | -1.986 | 0.047 |
| **Fertilization Parameters** |  |  |  |  |  |
| Normal fertilization rate (%) |  |  |  |  |  |
| <60 | Ref |  |  |  |  |
| 60-69 | -0.185 | 0.59147 | 0.831(0.255-2.645) | -0.313 | 0.755 |
| 70-84 | -0.987 | 0.55369 | 0.373(0.121-1.082) | -1.783 | 0.075 |
| ≥85 | -0.137 | 0.53249 | 0.872(0.298-2.449) | -0.257 | 0.797 |
| **Transfer Parameters** |  |  |  |  |  |
| Embryo transfer strategy |  |  |  |  |  |
| Single embryo transfer (SET) | Ref |  |  |  |  |
| Double embryo transfer (DET) | 0.316 | 0.35543 | 1.371(0.683-2.763) | 0.889 | 0.374 |
| Sequential embryo transfer |  |  |  |  |  |
| Performed | Ref |  |  |  |  |
| Not performed | -0.398 | 0.65217 | 0.672(0.169-2.341) | -0.61 | 0.542 |
| Embryo transfer stage |  |  |  |  |  |
| Cleavage stage | Ref |  |  |  |  |
| Blastocyst | -0.995 | 0.57801 | 0.37(0.109-1.107) | -1.722 | 0.085 |
| Type of embryo transfer cycle |  |  |  |  |  |
| Fresh embryo transfer | Ref |  |  |  |  |
| Frozen embryo transfer | -0.521 | 0.36263 | 0.594(0.288-1.201) | -1.438 | 0.151 |
| Endometrial thickness on transfer day (mm) |  |  |  |  |  |
| ≤12 | Ref |  |  |  |  |
| ＞12 | -0.637 | 0.38401 | 0.529(0.246-1.118) | -1.66 | 0.097 |
| **Comorbid Conditions** |  |  |  |  |  |
| Tubal factor infertility | Ref |  |  |  |  |
| Present |  |  |  |  |  |
| Absent | 0.308 | 0.35803 | 1.361(0.674-2.756) | 0.861 | 0.389 |
| Adenomyosis |  |  |  |  |  |
| Present | Ref |  |  |  |  |
| Absent | -1.407 | 0.802 | 0.245(0.036-0.998) | -1.755 | 0.079 |
| Uterine fibroids |  |  |  |  |  |
| Present |  |  |  |  |  |
| Absent | -0.833 | 0.48759 | 0.435(0.157-1.092) | -1.709 | 0.087 |
| Asthenospermia |  |  |  |  |  |
| Present | Ref |  |  |  |  |
| Absent | 0.794 | 0.43397 | 2.213(0.955-5.308) | 1.83 | 0.067 |
| **Endometrioma Characteristics** |  |  |  |  |  |
| Cyst diameter (cm) |  |  |  |  |  |
| 4-5 | Ref |  |  |  |  |
| 5-6 | 2.343 | 0.54292 | 10.41(3.764-32.17) | 4.316 | 0 |
| ＞6 | 2.708 | 0.5164 | 15(5.733-44.15) | 5.244 | 0 |
| Cyst recurrence status |  |  |  |  |  |
| Primary | Ref |  |  |  |  |
| Recurrent | 0.26 | 0.4508 | 1.297(0.541-3.22) | 0.576 | 0.564 |
| Cyst laterality |  |  |  |  |  |
| Unilateral | Ref |  |  |  |  |
| Bilateral | -0.158 | 0.41991 | 0.854(0.373-1.959) | -0.377 | 0.706 |
| Cyst number |  |  |  |  |  |
| Single | Ref |  |  |  |  |
| Multiple | 0.693 | 0.3963 | 2(0.933-4.45) | 1.749 | 0.08 |

Supplementary Table S1. Univariate Logistic Regression Analysis Results for All Candidate Variables. Note: Data are presented as beta coefficient (β), standard error (SE), odds ratio (OR) with 95% confidence interval (CI), Z-statistic, and P-value. BMI, Body Mass Index; bFSH, Basal Follicle-Stimulating Hormone; bLH, Basal Luteinizing Hormone; bE_2_, Basal Estradiol; bT, Basal Testosterone; bP, Basal Progesterone; bPRL, Basal Prolactin; COH, Controlled Ovarian Hyperstimulation; GnRH, Gonadotropin-Releasing Hormone; PPOS, Progestin-Primed Ovarian Stimulation; IVF, In Vitro Fertilization; ICSI, Intracytoplasmic Sperm Injection; HCG, Human Chorionic Gonadotropin; MII, Metaphase II oocytes; SET, Single Embryo Transfer; DET, Double Embryo Transfer; Ref, Reference category.
